# Drug resistant* Mycobacterium tuberculosis* in Oman: resistance-conferring mutations and lineage diversity

**DOI:** 10.7717/peerj.13645

**Published:** 2022-07-28

**Authors:** Sara Al Mahrouqi, Amal Gadalla, Saleh Al Azri, Salama Al-Hamidhi, Amina Al-Jardani, Abdullah Balkhair, Amira Al-fahdi, Laila Al Balushi, Samiya Al Zadjali, Asmahan Mohammed Nasser Al Marhoubi, Hamza A. Babiker

**Affiliations:** 1 Biochemistry Department, College of Medicine and Health Sciences, Sultan Qaboos University, Oman, Muscat, Oman; 2 Division of Population Medicine, School of Medicine, College of Biomedical Sciences, Cardiff University, Cardiff, United Kingdom; 3 Central Public Health Laboratories, MOH, Muscat, Oman; 4 Department of Medicine, College of Medicine and Health Sciences, Sultan Qaboos University, Oman, Muscat, Oman; 5 National Tuberculosis Reference Laboratory, MOH, Muscat, Oman; 6 Institute of Immunology and Infection Research, School of Biological Sciences, University of Edinburgh, Edinburgh, United Kingdom

**Keywords:** *Mycobacterium tuberculosis*, Drug resistance genes, Spoligotypes, Oman

## Abstract

**Background:**

The Sultanate of Oman is country a low TB-incidence, with less than seven cases per 10^5^ population detected in 2020. Recent years have witnessed a persistence in TB cases, with sustained incidence rate among expatriates and limited reduction among Omanis. This pattern suggests transmission from the migrant population. The present study examined the genetic profile and drug resistance-conferring mutations in *Mycobacterium tuberculosis* collected from Omanis and expatriates to recognise possible causes of disease transmission.

**Methods:**

We examined *M. tuberculosis* cultured positive samples, collected from Omanis (*n* = 1,344) and expatriates (*n* = 1,203) between 2009 and 2018. These isolates had a known *in vitro* susceptibility profile to first line anti-TB, Streptomycin (SM), Isoniazid (INH), Rifampicin (RIF), Ethambutol (EMB) and Pyrazinamide (PZA). The diversity of the isolates was assessed by spacer oligo-typing (spoligotyping). Drug resistance-conferring mutations resulted from full-length sequence of nine genes (*katG, inhA, ahpc, rpoB, rpsL, rrs, embB, embC, pncA*) and their phenotypic relationship were analysed.

**Results:**

In total, 341/2192 (13.4%), *M. tuberculosis* strains showed resistance to any drug, comprising mono-resistance (MR) (242, 71%), poly-resistance (PR) (40, 11.7%) and multi-drug resistance (MDR) (59, 17.3%). The overall rate of resistance among Omanis and expatriates was similar; however, MDR and PZA^R^ were significantly higher among Omanis, while INH^R^ was greater among expatriates. Mutations *rpsL* K43R and* rpoB* S450L were linked to Streptomycin (SM^R^) and Rifampicin resistance (RIF^R^) respectively. Whereas, katG S315T and *inhA* –C15T/G–17T were associated with Isoniazid resistance (INH^R^). The resistance patterns (mono-resistant, poly-resistant and MDR) and drug resistance-conferring mutations were found in different spoligo-lineages. *rpsL* K43R, *katG* S315T and *rpoB* S450L mutations were significantly higher in Beijing strains.

**Conclusions:**

Diverse drug resistant *M. tuberculosis* strains exist in Oman, with drug resistance-conferring mutations widespread in multiple spoligo-lineages, indicative of a large resistance reservoir. Beijing’s *M. tuberculosis* lineage was associated with MDR, and multiple drug resistance-conferring mutations, favouring the hypothesis of migration as a possible source of resistant lineages in Oman.

## Introduction

Oman is a low burden tuberculosis (TB) country with an incident rate of seven per 100,000 population in 2020 (WHO, 2021). The disease is widespread, with equal risk among nationals and expatriates (Al Abri et al., 2020a; Al Abri et al., 2020b; Hegazy et al., 2021), who represent a 45% of the population, with the vast majority from the Indian subcontinent (NCSI, 2020). The initial phase of the national control program resulted in a substantial reduction in TB cases (73%) between 1981 and 1992, with an average reduction of 9% per year (Al-Maniri et al., 2010; Al Awaidy, 2018). However, recently the TB reduction rates have slowed to 4.6% per year, with a large proportion of new TB cases seen among expatriates (Al Abri et al., 2020a; Al Abri et al., 2020b; Al Awaidy, 2018). For example, 8.1, 7.7, 6.3, 4.7 and 3.3 new TB cases were seen among Omanis compared to 9.8, 8.1, 7.7, 7.1 and 7.3 per 100,000 population in expatriates in 2014, 2015, 2016, 2017 and 2018, respectively. The vast majority of infected expatriates were from high TB burden countries in the Indian subcontinent, including India (721 (76%)), Bangladesh (103 (11%)) and Pakistan (25 (3%)) (Al Awaidy, 2018).

The increasing pattern of TB cases among expatriates in Oman (Al Abri et al., 2020a; Al Abri et al., 2020b) may reflect *M. tuberculosis* transmission of reactivated latent TB (LTB) (Aldridge et al., 2018), or transmission due to poor social conditions (*e.g.*, overcrowding and poor living conditions). This can be exacerbated by the fact that some expatriates seek medical advice at an advanced stage of the disease (Al Mayahi et al., 2020). Consequently, TB incidence rates among expatriates based on case notification may be lower than anticipated (Pandey et al., 2017; Romanowski et al., 2019; Snow et al., 2018). Thus, TB control targets in Oman for 2035 and the elimination threshold of 1 per million population (Lönnroth et al., 2015) may not be met without a clear information on the impact of expatriates and whether they serve as a transmission reservoir of drug resistant strains. A significant proportion of drug resistant cases, particularly multi-drug resistant (MDR), in migrants from high burden countries to Europe had resulted from reactivation of LTB (Hargreaves et al., 2017). Therefore, the high influx of expatriates from TB endemic areas poses a great risk for the spread of drug resistance in Oman.

The present study investigated the characteristics and diversity of drug resistant *M. tuberculosis* strains in Oman. We examined nine genes implicated in resistance to first-line anti-TB therapy (isoniazid (INH), rifampicin (RIF), ethambutol (EMB), streptomycin (SM) and pyrazinamide (PZA)). Known mutations in these genes are associated with resistance of *M. tuberculosis* to drugs such as SM, INH and RIF (Iacobino, Fattorini & Giannoni, 2020). However, due to the global geographical diversity of *M. tuberculosis*, and the high proportion of expatriates in Oman, there is a possibility of varying efficacy of these mutations, which could influence their diagnostic value. Thus, it is important to validate the role of these mutations in Oman and examine their spatiotemporal distribution among Omanis and expatriates. This information can lead to optimization of current management and control strategies to limit the evolution of drug resistance.

## Materials and Method

### Study population and characteristic of *M. tuberculosis* isolates

A total of 2,547 unique *M. tuberculosis* isolates were obtained from pulmonary and extra-pulmonary TB diagnosed Omanis (*n* = 1, 344) and expatriates (*n* = 1, 203), between 2009 and 2018 at the Central Public Health Laboratories (CPHL), Ministry of Health, Oman. The laboratory provides TB diagnosis, and *in vitro* drug susceptibility testing (DST) for the first line anti-TB drugs, to the population of Oman (4.4 million people comprising 2.8 nationals and 1.6 expatriates). The largest proportion of expatriates in Oman are from high TB burden countries such as India, Pakistan and Bangladesh (NCSI, 2020).

### Microbiology methods

Standard microbiological assays were carried out by staff at CPHL, for the identification of members of the *M. tuberculosis* complex. DST was performed on 2,539 isolates (SM, 1.0 µg/ml; INH, 0.1 µg/ml; RIF, 1.0 µg/ml; EMB, 5.0 µg/ml; PZA, 100.0 µg/ml) using the BACTEC MGIT 960 system (Ardito et al., 2001). A sensitive response refers to susceptibility to all first line drugs (SM, INH, RIF, EMB, and PZA), whereas resistance to any drugs refers to growth at or above the lowest drug concentration that inhibits the wild type (susceptible) strain. Different drug resistance profiles were identified including: resistance to a single drug (mono-resistant (MonoR)), resistance to more than one drug but not to both INH and RIF (poly-resistant (PolyR)), resistance to at least INH plus RIF (multi-drug resistant (MDR)),) and MDR plus resistance to a second line anti-TB treatment, fluoroquinolone, and least one of the injectable second-line drugs (extensively-drug resistant (XDR)) (Song et al., 2019).

Ethical approval (SQU-EC/075/18) for the study was granted by the Ethics Committee of The College of Medicine and Health Sciences, Sultan Qaboos University, Oman. Patients’ consent was not obtained as this study examined archived heat killed DNA samples obtained from material cultured as part of the routine diagnosis process by staff of the Central Public Health Laboratories, Ministry of Health, Oman.

### Detection of drug resistance-conferring mutations

DNA was extracted from *Mycobacterium tuberculosis* isolates using the heat-killing method (Warren et al., 2006). PCR and targeted sequencing were used to detect mutations in nine genes implicated in resistance to SM (*rpsL* and *rrs*), INH (*katG*, *ahpc*, *InhA/InhA* promoter), RIF (*rpoB*), PZA (*pncA*) and EMB (*embB* and *emb* C). Primer sequences and PCR conditions are described in Table S1.

### Spoligotyping

Spacer oligonucleotide typing (spoligotyping) was successfully completed on 1,295 isolates (629 Omanis and 666 expatriates), as described elsewhere (Al-Mahrouqi et al., 2022; Kamerbeek et al., 1997). Briefly, the direct repeat (DR) region of *M. tuberculosis* was amplified using a pair of PCR primers. The PCR products were hybridized to a set of 43 oligonucleotide probes corresponding to spacers on the DR region and covalently bound to the membrane. Obtained spoligo-patterns were then compared to the SITVIT-Web database (http://www.pasteur-guadeloupe.fr:8081/SITVIT_ONLINE, http://www.pasteur-guadeloupe.fr:8081/SITVIT2/files/SITVIT-KBBN_report_310313.xls) for identification of spoligo families and spoligotype international types (SIT) (Demay et al., 2012).

### Data analysis

Associations between categorical variables were tested using Chi-squared and/or Fisher’s exact tests and was applied to the association of DST among isolates from Omanis and expatriates, as well as the association of drug resistance-conferring mutations with their corresponding DST. In addition, chi-squared/Fisher’s exact tests were used to examine the distribution of different resistance profiles (MonoR, PolyR and MDR) and drug resistance-conferring mutations in different *M. tuberculosis* lineages. Fisher’s exact test was used to compare variables when the observed count was <5 in 20% or more of the cells in 2 × 2 chi-squared table. Logistic regression models were used to estimate the effect of demographic variables (Omanis vs expatriates) on the probability of detecting DST. Odds ratio (OR) and 95% confidence intervals (CI), with their associated log likelihood chi-squared test were presented for logistic regression models. Analyses was conducted in SPSS, version 23 and R program, version 1.4.1717.

## Results

### Characteristics of study subjects

We examined 2,539 of 2,547 *M. tuberculosis* strains obtained from Omanis (1,341, 52.8%) and expatriates (1,203, 47.2%). The age of patients ranged between 3 and 100 years, with a mean of 44.2 (20.2 ± SD) among Omanis and 33.7 (10.2 ± SD) for expatriates. Most of the patients were male (*n* = 1, 549, (61.0%)) with a slightly higher percentage of males among expatriates (51.8%) compared to Omanis (48.2%) (*P* = 0.218).

### Drug susceptibility profile of *M. tuberculosis* isolates

Of the 2,539 *M. tuberculosis* isolates examined, 2,198 (86.6%) were sensitive to the first line therapy (INH, RIF, SM, EMB and PZA) (Fig. S1). The remaining 341 (13.4%) isolates fell into four drug resistance profiles: 242 (71%) were MonoR, 40 (11.7%) were PolyR, 49 (14.4%) were MDR, and 9 (2.6%) wereXDR. Of the MonoR strains, 92 (38%) were INH^R^, 76 (31.4%) were PZA^R^, 65 (26.9%) were SM^R^ and 9 (3.7%) were RIF^R^ (Table 1).

**Table 1:** Drug response profile of *M.tuberculosis* isolates obtained from Omanis and expatriates, between 2009 and 2018.

	Expatriates 1,198 (47.2%)	Omanis 1,341 (52.8%)	*P-value*
Sensitive	1022 (85.3%)	1176 (87.6%)	0.637^*^
Any drug resistance	176 (14.7%)	165 (12.3% )	
SM^R^	35 (53.8%)	30 (46.2%)	0.689^a^
INH^R^	60 (65.2%)	32 (34.8%)	0.002^a^
RIF^R^	4 (44.4%)	5 (55.6%)	0.663^a^
PZA^R^	31 (40.8%)	45 (59.2%)	0.032^a^
PolyR	25 (62.5%)	15 (37.5%)	0.143^a^
MDR	21 (35.6%)	38 (64.4%)	0.007^a^

**Notes.**

* *χ*2.

a logistic regression.

SM^R^ Streptomycin mono-resistant INH^R^ Isoniazid mono-resistant RIF^R^ Rifampicin mono-resistant PZA^R^ Pyrazinamide mono-resistant PolyR Poly-resistant MDR Multi-drug-resistant

Similar rate of resistance to any drug was seen among *M. tuberculosis* isolated from Omanis and expatriates (*P* = 0.637). However, variations were noted in INH^R^ (Log Likelihood ratio = −194.30; *χ*2, 9.66; *P* = 0.002), PZA^R^ (Likelihood ratio = −179.9; *χ*2,5.01; *P* = 0.025) and MDR (Log Likelihood ratio = −140.16; *χ*2,4.275; *P* = 0.039). INH^R^ was lower among Omanis (OR = 0.46; 95% CI [0.28–0.75]), contrasting the elevated rates of PZA^R^-TB (OR = 1.79; 95% CI [1.07–3.02]) and MDR (OR = 1.90; 95% CI [1.03–3.56]) (Table 2).

**Table 2:** Drug resistance response profiles among different *M.tuberculosis* obtained from Omanis and expatriates.

	**Odds ratio**	**95% CI** ^a^	
		**Lower**	**Upper**
**INH** ^**R**^ **-TB** ^b^			
Non-Omani (*n* = 60)	Referent		
Omani (*n* = 32)	0.46	0.28	0.75
**MDR-TB** ^c^	
Non-Omani (*n* = 21)	Referent		
Omani (*n* = 38)	1.90	1.03	3.56
**PZA** ^**R**^ **-TB** ^d^			
Non-Omani (*n* = 31)		Referent	
Omani (*n* = 45)	1.79	1.07	3.02

**Notes.**

a Logit regression.

b Isoniazid mono-resistant tuberculosis.

c Multi-drug resistant tuberculosis.

d Pyrazinamide mono-resistant tuberculosis.

### Spatial distribution of drug susceptibility in *M. tuberculosis*

**Figure 1:**
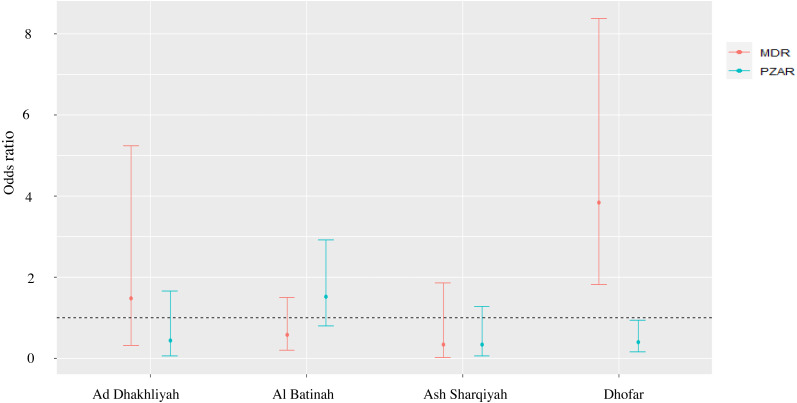
Odds ratio and 95% confidence interval of PZA^R^-TB and MDR-TB to evaluate the strength of association between DST profiles and provinces with > 10 reported TB cases.

The MonoR forms, SM^R^ (Log Likelihood ratio = −131.77; *χ*^2^,6.19; *P* = 0.186), INH^R^ (Log Likelihood ratio = −172.04; *χ*^2^,8.51; *P* = 0.074) and the PolyR cases (Log Likelihood ratio = −104.82; *χ*^2^,7.92; *P* = 0.095) were stable in different regions in Oman. However, variation was seen in RIF^R^ (Log Likelihood ratio = −31.17; *χ*2,10.88; *P* = 0.028), PZA^R^ (Log Likelihood ratio = −143.94; *χ*^2^,13.11; *P* = 0.011) and MDR (Log Likelihood ratio = −116.82; *χ*^2^,23.35; *P* = 0.000). For example, RIF^R^ was 14 and 11 times higher in Ad Dakhliyah (OR = 14.53; 95% CI [1.32–324.20]) and Ash Sharqiyah (OR = 11.47; 95% CI [1.05–254.22]), respectively, compared to Muscat. Likewise, in Dhofar, MDR was four time higher (OR = 3.84; 95% CI [1.82–8.38]) and PZA^R^ was significantly lower compared to Muscat (OR = 0.40; 95% CI [0.15–0.93]) (Fig. 1).

### Temporal distribution of drug susceptibility in *M. tuberculosis*

**Figure 2:**
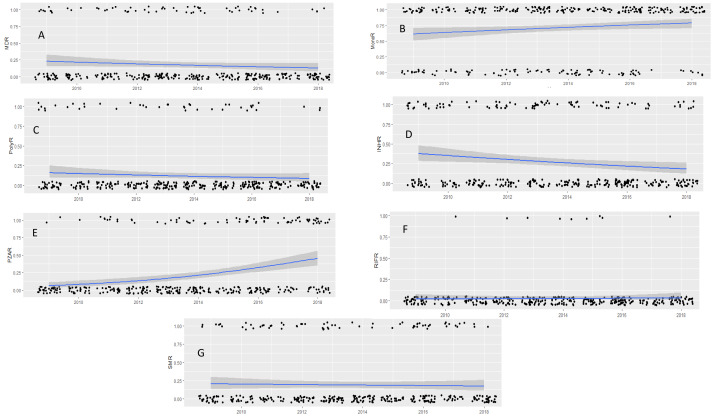
Drug susceptibility test (DST) profiles of *M. tuberculosis* isolates collected between 2009 and 2018. MDR pattern (A), mono-resistance (B), poly-resistance (C) and pattern of INH^R^ (D), PZA^R^ (E), RIF^R^ (F) SM^R^ (G). Black dots are the observed data with 0 being negative and 1 being positive for drug resistance response. Lines represent estimated prevalence overtime based on logistic regression models, gray shade around the line is the 95% confidence intervals. P and logistic regression model and odd ratio (OR) correspond to time of significant change in prevalence compared to the reference year (2009).

Overall, resistant *M. tuberculosis* isolates remained stable over the study period, with the exception of INH^R^ (Table S2). The PolyR and MDR *M. tuberculosis* strains remained stable over the study period (*P* = 0.158 and 0.248 respectively) (Figs. 2A & 2B). However, the MonoR strains increased steadily, with 2 times higher prevalence in 2018 compared to 2009 (Log Likelihood = −791.78; *χ*2,19.98; *P* = 0.025) (OR 2.1; 95% CI [1.13–3.76]) (Fig. 2C), driven by PZA^R^ (Log Likelihood = −161.4; *χ*^2^,42.00; *P* = 0.000) (Fig. 2D). In contrast, INH^R^ decreased steadily (Log Likelihood = −189.7; *χ*^2^,19.00; *P* = 0.025) (Fig. 2E) while SM^R^ (*P* = 0.113) and RIF^R^ (*P* = 0.210) remained unchanged (Figs. 2F & 2G).

### Diversity of drug resistant *M. tuberculosis* isolates

Spoligotyping was completed for 242/341 (70.9%) drug-resistant *M. tuberculosis* isolates (106 Omanis and 136 expatriates) (Fig. S1). Of these, 187 (76.4%) exhibited known spoligotype patterns (SIT), while 55 (22.7%) were ‘orphans’. The isolates with known spoligotypes comprised 8 lineages, EAI (66, 35.3%), CAS (50, 26.7%), Beijing (28, 15.0%), T (21, 11.2%), LAM (10, 5.3%), H (5, 2.7%), X (2, 0.5%), Ural-1 (2, 0.5%), Cameroon (2, 0.5%) and UK (1, 0.5%). Of these, 137 (73.26%) isolates existed in clusters of 33 spoligo-profiles (with 2 to 28 isolates), and 50 (26.74%) were singletons, each with a distinct profile. Of the above clusters, 16/33 (48.48%) included *M. tuberculosis* isolates from both Omanis and expatriates (Table 3), suggesting close genetic relatedness and possible transmission of drug-resistant lineages between the two groups.

**Table 3:** Distribution of clustered drug resistant *M tuberculosis* lineages among Omanis and expatriates.

Clade	SIT	Expatriate n(%)	Omani n(%)	Total n(%)	*P*-value
Beijing	1	17 (36.2)	11 (22.0)	28 (28.9)	0.124
CAS1-Delhi	25	2 (4.2)	6 (12.0)	8 (8.2)	0.526
	26	12 (25.5)	12 (24.0)	24 (24.7)	
	309	1 (2.1)	2 (4.0)	3 (3.1)	
EAI1-SOM	48	2 (4.2)	1 (2.0)	3 (3.1)	0.758
	72	1 (2.1)	3 (6.0)	4 (4.1)	
EAI3-IND	11	3 (6.4)	1 (2.0)	4 (4.1)	0.918
	298	1 (2.1)	1 (2.0)	2 (2.1)	
	3803	1 (2.1)	3 (6.0)	4 (4.1)	
EAI5	126	1 (2.1)	1 (2.0)	2 (2.1)	ND^*^
	2921	1 (2.1)	2 (4.0)	3 (3.1)	
LAM11-ZWE	59	1 (2.1)	2 (4.0)	3 (3.1)	ND
T1	393	1 (2.1)	1 (2.0)	2 (2.1)	ND
T2	52	1 (2.1)	1 (2.0)	2 (2.1)	ND
T3-ETH	149	1 (2.1)	2 (4.0)	3 (3.1)	ND
X3	92	1 (2.1)	1 (2.0)	2 (2.1)	ND
Total		47	50	97	

**Notes.**

* not done.

The patterns of resistance (MonoR, PolyR and MDR) were found in most of the spoligotype-defined lineages (Table 4). However, MonoR and MDR were significantly associated with strain lineage (*p* = 0.000) for both types of resistance patterns (Table 3). MonoR was disproportionately higher in the EAI and CAS lineages, whereas MDR was over-represented in the Beijing, CAS, and T lineages. The variation in MonoR seen in the different linages was linked to SM^R^ (*p* > 0.001) and PZA^R^ (*p* > 0.001) (Table 4).

**Table 4:** Distribution of drug resistance profiles among *M.tuberculosis* sub-lineages in Oman, 2014–2018.

Clade	Total	Any drug resistance			Total	Resistance to one drug			
		*n* = 242				**n=181**			
		**MonoR** ^b^	**PolyR** ^c^	**MDR** ^d^		**SMR** ^e^	**INHR** ^f^	**RIFR** ^g^	**PZAR** ^h^
EAI	100	90(37.2%)	8(3.3%)	2(0.8%)	90	13(7.2%)	23(12.7%)	1(0.6%)	53(29.3%)
BEIJING	28	12(5.0%)	4(1.7%)	12(5.0%)	12	8(4.4%)	3(1.7%)	–	1(0.6%)
CAS	60	41(16.9%)	9(3.7%)	10(4.1%)	41	15(8.3%)	19(10.5%	4(2.2%)	3(1.7%)
T	25	13(5.4%)	2(0.8%)	10(4.1%)	13	2(1.1%)	7(3.9%)	2(1.1%)	2(1.1%)
LAM	11	8(3.3%)	3(1.2%)	–		1(0.6%)	6(3.3%)	–	1(0.6%)
H	10	10(4.1)	–	–	10	3(1.7%)	6(3.3%)	–	1(0.6%)
Others^a^	8	25(10.3%)	3(1.2%)	1(0.4%)	25	6(3.3%)	12(6.6%)	1(0.6%)	6(3.3%)
*P-* value		0.000	0.154	0.000		0.028	0.632	0.329	0.000

**Notes.**

a Cameroon (*n* = 2), Manu (*n* = 2), X(*n* = 3), Zero (*n* = 1).

b Mono-drug resistant.

c Poly-drug resistant.

d Multi-drug resistant.

e Streptomycin mono-resistant.

f Isoniazid mono-resistant.

g Rifampicin mono-resistant.

h Pyrazinamide mono-resistant.

### Mutations in *M. tuberculosis* drug resistance genes

Full length sequences for nine genes implicated in resistance to SM (*rpsL* and *rrs*), INH (*katG*, *ahpc*, *InhA/InhA* promoter), RIF (*rpoB*), PZA (*pnc* A) and EMB (*emb* B and *emb* C) were obtained for 356 of the *M. tuberculosis* isolates (211 (58.8%) sensitive and 154 (42.2%) resistant to any drug) (Fig. S1). The resistant isolates were obtained from 58 (37.7%) Omanis and 96 (62.3%) expatriates (Table 5).

**Table 5:** Drug resistance conferring mutations among *M.tuberculosis* in Oman, 2009 to 2018.

Drug	Gene	Allele	Overall mutation prevalence	Sensitive isolates mutation (%)	Resistant isolates mutation (%)	*p-* value
Streptomycin	*rpsL*	K43R	20/282(7.1%)	1/240 (0.4%)	19/42 (45.2%)	0.000
		R86W	1/282(0.4%)	0.00 (0.00%)	1/42 (2.4%)	ND^*^
		V19F	1/282(0.4%)	1/240 (0.4%)	0(0%)	ND
	*rrs*	Q302E	2/190(1.1%)	1/168 (0.6%)	1/22 (4.5%)	ND
		L341F	3/190(1.6%)	3/168 (1.8%)	(0%)	ND
		A409G	1/190(0.5%)	1/168 (0.6%)	(0%)	ND
Isoniazid	*inhA,* (Pro)	C-15T	14/299(4.7%)	1/233 (0.4%)	13/66 (19.7%)	0.000
		G-17T	7/299(2.3%)	(0%)	7/66 (10.6%)	0.000
		G-47A	1/299(0.3%)	1/233 (0.4%)	(0%)	ND
	*inhA, (OF)*	I95L	1/313(0.3%)	(0%)	1/71 (1.4%)	ND
		I194T	1/313(0.3%)	(0%)	1/71 (1.4%)	ND
	*katG*	S315T	34/309(11%)	1/239 (0.4%)	33/70 (47.1%)	0.000
		P364S	2/298(0.7%)	1/239 (0.4%)	1/70 (1.4%)	ND
		A379D	1/298(0.3%)	(0%)	1 (0.3%)	0.228
		S383A	1/298(0.3%)	1/239 (0.4%)	(0%)	ND
		R463L	166/298(55.7%)	129/239 (54.0%)	37/70 (52.9%)	0.457
		L472I	1/298(0.3%)	1/239 (0.4%)	(0%)	ND
		T475I	1/298(0.3%)	1/239 (0.4%)	(0%)	ND
		V503A	2/298(0.7%)	2/239 (0.8%)	(0%)	ND
		L526H	1/298(0.3%)	(0%)	1/70 (1.4%)	ND
		D735A	1/298(0.3%)	(0%)	1/70 (1.4%)	ND
	*ahpC*	F77V	1/250(0.4%)	1/194 (0.5%)	(0%)	ND
Rifampicin	*rpoB*	H343Q	1/306(0.3%)	1/276 (0.4%)	(0%)	ND
		V359A	1/306(0.3%)	1/276 (0.4%)	(0%)	ND
		M390T	1/306(0.3%)	1/276 (0.4%)	(0%)	ND
		S441L	1/306(0.3%)	(0%)	1/30 (3.3%)	ND
		H445L	1/306(0.3%)	(0%)	1/30 (3.3%)	ND
		H445D	1/306(0.3%)	(0%)	1/30 (3.3%)	ND
		S450L	16/306(9.8%)	(0%)	16/30 (53.3%)	0.000
		K512R	8/306(2.6%)	7/276 (2.5%)	1/30 (3.3%)	ND
		P541L	1/306(0.3%)	1/276 (0.4%)	(0%)	ND
		V575I	2/306(0.7%)	2/276 (0.8%)	(0%)	ND
Ethambutol	*embB*	M306V	4/287(1.4%)	3/281 (1.1%)	1/6 (16.7%)	ND
		M306I	2/287(0.7%)	2/281 (0.7%)	(0%)	ND
		A313V	1/287(0.3%)	1/281 (0.4%)	(0%)	ND
		S347R	1/287(0.3%)	1/281 (0.4%)	(0%)	ND
		E378A	111/287(38.7%)	111/287	(0%)	ND
		G406D	2/287(0.7%)	2/281 (0.7%)	(0%)	ND
	embC	T270I	41/101(40.6%)	41/98 (41.8%)	(0%)	ND
		N394D	40/101(39.6%)	40/98 (40.8%)	(0%)	ND
Pyrazinamide	*pncA*	I6L	1/287(0.3%)	(0%)	1/54 (1.9%)	ND
		Q10^*^	1/287(0.3%)	(0%)	1/54 (1.9%)	ND
		D12A	2/287(0.7%)	(0%)	2/54 (3.7%)	ND
		D12E	1/287(0.3%)	(0%)	1/54 (1.9%)	ND
		P54Q	2/287(0.7%)	1/233 (0.4%)	1/54 (1.9%)	ND
		H71R	1/287(0.3%)	(0%)	1/54 (1.9%)	ND
		F106L	1/287(0.3%)	1/233 (0.4%)	(0%)	ND
		G132D	1/287(0.3%)	(0%)	1/54 (1.9%)	ND

**Notes.**

* not done.

#### Streptomycin related mutations

Three mutations were detected in the *rpsL* sequences from 282 isolates*.* Of these, mutations V19F and R86W were rare, and seen among both streptomycin sensitive (SM^S^) and streptomycin resistant (SM^R^) isolates. However, mutation K43R was frequent, found in 45% of SM^R^ isolates compared to 0.4% SM^S^ isolates (*p* > 0.001) (Table 5). However, the *rrs* sequences (*n* = 190 isolates) showed three infrequent mutations (Q302E (1.1%), L341F (1.6%), A409G (0.5%)), seen among both SM^S^ and SM^R^ isolates (Table 5).

#### INH related mutations

*katG, ahpC, inhA* and its promoter region were screened for mutations conferring isoniazid resistance (INH^R^). Out of 298 isolates, 209 (70.1%) carried 10 mutations in *katG*, all at a low prevalence (<2%), with the exception of *katG* S315T (11%) and *katG* R463L (55.7%). The *katG* S315T mutation occurred in 47.1% of INH^R^ compared to 0.4% isoniazid sensitive (INH^S^) isolates (*p* > 0.001), whereas R463L was similar in INH^S^ (54.0%) and INH^R^ (52.9%) isolates (*P* = 0.457) (Table 5).

Mutations *inhA* promoter C-15T and G-17T were found in 19.7% (*p* > 0.001) and 10.6% (*p* > 0.001) of INH^R^ isolates compared to 0.4% and 0.00% of INH^S^ isolates, respectively. Additional rare mutations (I95L (1.4%) and I194T (1.4%)) were seen in the coding region of *inhA*,whereas no mutations were found in the *ahpC* gene (Table 5).

#### Rifampicin related mutations

*rpoB* held 10 mutations. Of these, S450L was linked to rifampicin resistance (RIF^R^) in 16/30 (53%) isolates (*p* > 0.001) (Table 3). This mutation plus a further 3 (S441L, H4445l, H445D) existed exclusively in RIF^R^ isolates, in the 81-bp rifampicin-resistance-determining region (RRDR) (codons 426–452), which is associated with high-level RIF resistance (Hirani et al., 2020). The remaining six mutations were rare, located outside the RRDR region, and seen only in rifampicin sensitive (RIF^S^) isolates, (Table 5).

#### Ethambutol related mutations

*emb* B (*n* = 287) and *emb* C (*n* = 101) harboured six and two mutations, respectively. With the exception of one mutation (*emb* B M306V/I), other mutations on both genes existed exclusively in ethambutol sensitive (EMB^S^) isolates. The *emb* B M306V/I mutation occurred at a substantially higher frequency among ethambutol resistant (EMB^R^) isolates (16.7%) compared to EMB^S^ isolates (1.1%) (Table 5). This mutation has been found to be associated with two- and ten-fold higher EMB MIC when the wild type was replaced with M306I and M306V, respectively (Starks et al., 2009). However, mutations *embB E* 378A (38.7%), *emb* C T270I (40.6%) and *emb* C N394D (39.6%), were common in EMB^S^, and are suggested to be ancestral *M. tuberculosis* markers (Brossier et al., 2015).

#### Pyrazinamide related mutations

Eight mutations detected in *pncA* occurred at low frequency (0.3 to 0.7%). These mutations were seen in 8/54 (14.8%) pyrazinamide resistant (PZA^R^) isolates compared to 2/287 (0.7%) of pyrazinamide sensitive (PZA^S^) isolates (*p* > 0.05) (Table 5).

### Drug resistance-conferring mutations among Omanis and expatriates

**Figure 3:**
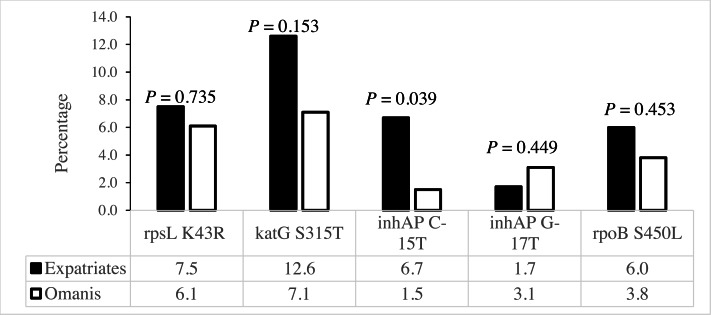
Distribution of drug resistance-conferring mutations among *M. tuberculosis* obtained from Omanis and expatriates.

The *rpsL* K43R, *katG* S315T and *rpoB* S450L mutations associated with SM^R^, INH^R^ and RIF^R^, respectively, existed at similar frequencies among Omanis and expatriates (Fig. 3). However, the C-15T mutation in the *inhA* promoter linked to INH^R^ existed at a significantly higher prevalence among expatriates compared to Omanis (Fig. 3).

### Drug resistance-conferring mutations in different *M. tuberculosis* lineages

We examined whether the distribution of the drug resistance-conferring mutations were influenced by *M. tuberculosis* genetic background. The *rpsL* K43R, *katG S* 315T and *rpoB* S450L mutations associated with SM^R^, INH^R^ and RIF^R^, respectively (Table 6), existed at different frequencies in different lineages (Table 6). The *rpsL* K43R, *katG* S315T and *ropB* S450L mutations were disproportionately higher in isolates of the Beijing lineage, whereas *katG* S315T and *inhA* C-15T mutations were over-represented in the EAI lineage. In contrast, isolates from the CAS lineage harboured all of the above mutations (Table 6). Interestingly, the orphan isolates which most likely represent locally evolved strains, had high rates of the evolutionary mutations, *embB* E378A, *embC* T270I, *embC* N394D and *katG* R463L (Table 6).

**Table 6:** Distribution of drug resistance conferring mutations among different *M. tuberculosis* lineages.

Mutation	Clades								*P*-value^a^
	**EAI**	**Beijing**	**CAS**	**T**	**LAM**	**H**	**Others** ^b^	**Orphan**	
*rpsL K43R*	–	10(55.6%)	1(5.6%)	2(11.1%)	–	1(5.6%)	1(5.6%)	3(16.7%)	0.000
*katG*-S315T	4(12.5%)	9(28.1%)	9(28.1%)	8(25.0%)	1(3.1%)	–	1(3.1%)	–	0.001
*inhA*-15	4(57.1%)	–	1(14.3%)	–	–	–	–	2(28.6%)	0.846
*inhA*-17	–	–	3(100.0%)	–	–	–	–	–	0.000
*rpoB* S450L	–	6(40.0%)	2(13.3%)	6(40.0%)	–	–	1(6.7%)	–	0.000
*katG* R463L	49(31.6%)	20(12.9%)	35(22.6%)	2(1.3%)	–	–	3(1.9%)	48(29.7%)	0.000
*embB* E378A	36(63.2%)	–	–	–	–	–	–	21(36.8%)	0.000
*embC* T270I	15(68.2%)	–	–	–	–	–	–	7(31.8%)	0.000
*embC* N394D	15(68.2%)	–	–	–	–	–	–	7(31.8%)	0.000

**Notes.**

a Logit regression.

b Cameroon, Manu, Turkey, S and X clades.

## Discussion

This study has revealed widespread resistance to anti-TB drugs in Oman, comprising MonoR, PolyR and MDR. These variable phenotypic responses were consistent between 2009 and 2018, and spatially, across different provinces of Oman (Table S2 ). The drug resistant *M. tuberculosis* strains were linked to mutations implicated in SM^R^, INH^R^ and RIF^R^ and MDR (*rpsL* K43R, *katG* S315T, *InhA* -15/-17, *rpoB* S450L) in other endemic sites (Ghosh, N. & Saha, 2020).

The variable drug resistance patterns we observed in Oman were distributed across 83 *M. tuberculosis* spoligotype-defined lineages, highlighting a highly diverse reservoir of resistant strains. However, some patterns were overrepresented in certain strain lineages (Table 4). For example, MonoR was disproportionately higher in the EAI and CAS lineages, while MDR was higher in isolates from the Beijing lineage (Table 4). These findings are in line with reports that identified a higher proportion of MonoR (RIF^R^ and SM^R^) among CAS and T lineages in India and other countries of origin of expatriates (Siva Kumar et al., 2020). The over-representation of MDR in isolates of the Beijing lineage is consistent with many studies that associated this family with any resistance and MDR (Ding et al., 2017). Lineage 2 strains, which includes the Beijing lineage, are most often isolated in Southeast Asia, India and East Africa (Couvin, Reynaud & Rastogi, 2019; Panwalkar, Chauhan & Desikan, 2017). Therefore, the high prevalence of the Beijing lineage in Oman (15.1%), can probably be attributed to the large influx of imported TB cases from Southeast Asia, India and East Africa (Al Abri et al., 2020a; Al Abri et al., 2020b), in line with the high frequency of shared spoligotype-defined lineages between Omanis and expatriates (Table 3) (Al-Mahrouqi et al., 2022). Thus, the diversity of drug resistant *M. tuberculosis* strains in Oman can, in part, be attributed to regular influx of novel drug resistant lineages via expatriates. Therefore, control strategies that can limit transmission as well as drug pressure and thereby positive selection of drug resistant strains should be considered. Drug pressure may not only select resistant strains, but also may enhance transmission (Leung et al., 2013), as several studies have shown that drug resistant TB is dominated by a few highly transmissible closely related clades (Koch & Cox, 2020).

The present study reinforces the association of mutations *rpsL* K43R and *ropB* S450L with SM^R^ and RIF^R^, respectively, as well as *katG* S315T and *inhA* promoter -15/-17 with INH^R^. Our findings confirm the role of these mutations in the phenotypic response of *M. tuberculosis* to these drugs, as reported in other geographical regions (Singh et al., 2020). The frequency of these mutations varies in different geographical regions. Consequently, their diagnostic value can differ in different countries. This necessitates analysis of local frequencies of these mutations to estimate the predictive diagnostic accuracy of drug resistance and their efficacy in epidemiological surveillance. These markers *rpsL* K43R (45.2% SM^R^), *kat* G S315T (47.1% INH^R^), *inhA* -15/-17 (30.3% INH^R^) and *rpoB* S450L (53.3% RIF^R^) detected a large proportion of drug resistant *M. tuberculosis* isolates in Oman. This accords with the worldwide reported figures observed for phenotypic resistance associated with the above mutations to streptomycin, isoniazid and rifampicin (Hazbo et al., 2006; Torres et al., 2015).

In addition, combinations of mutations implicated in resistance to one drug, can enhance the detectability of resistance. In the current study, *inhA* promoter mutations -15/-17 in the absence of any *katG* mutation detected 21.9% of INH^R^ isolates, while *katG* 315T alone identified 46% of INH^R^ isolates, which increased to 68% when both *katG* S315T and *inhA* -15/-17 were taken together. Thus, these mutations can provide valuable diagnostic tools to mitigate the risk of treatment failure and evolution of drug resistance. Nonetheless, we identified a number of rare mutations in the above genes (Table 5), as seen in other geographical regions (Hazbo et al., 2006; Torres et al., 2015). Regular monitoring of these mutations is important as is watching for the emergence of new mutations given drug pressure is expected to be a major determinant in the selection and spread of drug resistance genes. Some evidence suggests that acquired drug resistance results in stepwise acquisition and consistent increase in mutations can lead to a gradual increase in resistance (Prasad, Gupta & Banka, 2018).

Similar to the drug response profiles, the drug resistance-conferring mutations were spread in all *M. tuberculosis* lineages (Table 6), demonstrating the diversity and multiple origins of drug resistant strains in Oman. We found that MDR strains are more likely to harbour mutations *rpsL K43* R, *katG* S315T and *ropB* S450L linked to SM^R^, INH^R^ and RIF^R^, respectively, and that these mutations existed at a significantly higher prevalence among isolates of the Beijing and CAS genotypes (Table 6). Mutations *katG* S315T and *ropB* S450L are known to be low-cost mutations, associated with high-level RIF^R^ and INH^R^ (Ding et al., 2017). This association has been explained by possible positive epistasis between the Beijing genetic background and drug-resistance-conferring mutations (Borrell & Gagneux, 2009), making it favourable for drug selection and dissemination of MDR. Therefore, the high prevalence of CAS (25.4%) and Beijing (15.1%) lineages among drug resistant *M. tuberculosis* strains in Oman, emphasises the epidemiologically importance of these lineages, and the spread of drug resistance via imported TB cases form the Indian Sub-continent, East Africa and Southeast Asia, where these lineages predominate (Cain et al., 2015).

An important limitation of the present study was the absence of a more sensitive *in vitro* drug test for the *M. tuberculosis* isolates. The phenotypic drug response was based on DST and not MIC, and therefore, it was not possible to relate mutations to different levels of drug resistance, as it has been suggested that different combination of mutations in *katG* and *inhA* can result in different MIC for INH (Lempens et al., 2018). Accurate phenotypic data is critical for the identification of highly sensitive and specific drug resistance markers.

The absence of discriminatory genotypic data impeded proper interpretation of genetic relatedness of INH *M. tuberculosis* haplotypes shared between Omanis and expatriates. Many mutant haplotypes belong to *M. tuberculosis* strains shared between Omanis and expatriates. In addition, further study using a more discriminatory technique such as MIRU-VNTR or whole genome sequencing can identify transmission routes of the selective drug resistant strains with shared spoligotypes.

In summary, the spread of drug resistant *M. tuberculosis* in Oman is linked to known drug resistance conferring mutations, supporting the value of these mutations as diagnostic and surveillance tools to limit the spread of drug resistance. The occurrence of these mutations in different spoligotype-defined lineages, is an indicative of a large reservoir of resistant strains in Oman. The most critical drug resistance mutations are over-represented in isolates of the Beijing lineage, reinforcing their value to predict MDR. The similar frequencies of drug resistance mutations among *M. tuberculosis* obtained from Omanis and expatriates highlights the need to bolster molecular surveillance of TB infection.

## Supplemental Information

10.7717/peerj.13645/supp-1 Supplemental Information 1 Primer sequence for amplifying of *M. tuberculosis* drug resistance genes

10.7717/peerj.13645/supp-2 Supplemental Information 2 *In vitro* drug susceptibility to the first line anti TB therapy among MTB isolates during 2009–2018

10.7717/peerj.13645/supp-3 Supplemental Information 3 Flowchart showing number of MTB isolates successfully analyzed at different stages of the study

10.7717/peerj.13645/supp-4 Supplemental Information 4 *In vitro* drug response of *M. tuberculosis* in Oman 2009 to 2019

10.7717/peerj.13645/supp-5 Supplemental Information 5 Raw data of drug resistance mutations in *M. tuberculosis* in Oman 2009 to 2019

## Additional Information and Declarations

### Data Availability

The following information was supplied regarding data availability: The raw data is available in the Supplementary Files.
