# Supplementary material for: Drug resistant Mycobacterium tuberculosis in Oman: resistance-conferring mutations and lineage diversity
Source: PeerJ. 2022 Jul 28;10:e13645. doi: 10.7717/peerj.13645 (PMC9339217; doi:10.7717/peerj.13645)
Supplement: Supplemental Information 1 [file peerj-10-13645-s001.pdf]

Supplementary Table 1: Primers sequence for amplifying of *M. tuberculosis* drug resistance genes.

**Drug target**	**Gene**	**Primer sequence**	**Fragment size**	**Target region**
STR	*rps*L	F-rpsL: 5’-GAATCGAGTTTGAGGCAAGC-3’<br>R-rpsL: 5’-TCCCCTTCAACAGAACCTTG-3’	583bp	Full length gene
	rrs-F1	F-rrs: 5’-AGGTGTGGGTTTCCTTCCTT-3’<br>R-rrs: 5’-AGTGTGTTGGTGGCCAACTT-3’	829bp	Loops 530 & 915
INH	*kat*G	F-kaG1: 5’-CTTTCTCCTCATCCCCGTCT-3’<br>R-kaG1: 5’-ATTCGCACCCTGGAAGAGAT-3’	843bp	Full length gene
		F-kaG2: 5’-CGCCTTTGCTGCTTTCTCTA-3’<br>R-kaG2: 5’-GCGGTCACACTTTCGGTAA-3’	872bp	
		F-kaG3: 5’-GTGCCATACGAGCTCTTCCA-3’<br>R-kaG3: 5’-CCCGATAACACCAACTCCTG-3’	948bp	
	*inh*A	F-PiA: 5’-GCGACATACCTGCTGCGCAA-3’<br>R-PiA: 5’-ATCCCCCGGTTTCCTCCGGT-3’	523bp	Promoter region
		F-inhA1: 5’-CTTCCGAGGATGCGAGCTAT-3’<br>R-inhA1: 5’-ACCGTCATCCAGTTGTAGGC-3’	577bp	Full length gene
		F-inhA2: 5’-CATCTCGGCGTATTCGTATG-3’<br>R-inhA2: 5’-ACCGAAATGCAGGTAGTGCT-3’	600bp	
	ahpC	F-inhA2: 5’-GCTCATATCGAGAATGCTTGC-3’<br>R-inhA2: 5’-GCGGCCTTGAGCTTTTCTAT-3’	849bp	
RIF	*rpo*B	F-rpoB1: 5’-GCAGACGCTGTTGGAAAACT-3’<br>R-rpoB1: 5’-GCTCCAGGAAGGGAATCATC-3’	920bp	Clusters I (including RRDR), II, III
		F-rpoB2: 5’-GAGCCAATTCATGGACCAG-3’<br>R-rpoB2: 5’-CGTTGTCGTGCATCACAGT-3’	686bp	
EMB	*emb*B	F-embB: 5’-TGACCGACGCCGTGGTGATA-3<br>R-embB: 5’-GCCATGAAACCGGCCACGAT-3	1312bp	ERDR
	embC	F-embC: 5’-GTGCTTGTGGTGCGTAATGT-3<br>R-embC: 5’-AGCACCAGGTCAGCAGGAT-3	844bp	
PZA	*pncA*	F-pncA 5′-CGGATTTGTCGCTCACTAC-3′<br>R-pncA 5′-GCCGGAGACGATATCCAGAT-3′	960bp	Full length gene
		R-tlyA 5′-CAGCAGAACACTGCGATGAG-3′		

STR (Streptomycin), INH (Isoniazid), RIF (Rifampicin), EMB (Ethambutol), PZAR (Pyrazinamide)
