# Supplementary material for: Drug resistant Mycobacterium tuberculosis in Oman: resistance-conferring mutations and lineage diversity
Source: PeerJ. 2022 Jul 28;10:e13645. doi: 10.7717/peerj.13645 (PMC9339217; doi:10.7717/peerj.13645)
Supplement: Supplemental Information 2 [file peerj-10-13645-s002.pdf]

Supplementary Table 2. *In vitro* drug susceptibility to the first line anti TB therapy among MTB isolates during 2009-2018

	2009	2010	2011	2012	2013	2014	2015	2016	2017	2018	Total	*P*-value
Sensitive	213<br>85.9%	203<br>89.0%	246<br>89.1%	255<br>88.5%	231<br>84.6%	246<br>86.6%	231<br>88.5%	219<br>82.3%	203<br>89.4%	151<br>80.3%	2198<br>86.6%	0.050
SMR	6<br>2.4%	5<br>2.2%	9<br>3.3%	5<br>1.7%	10<br>3.7%	4<br>1.4%	3<br>1.1%	12<br>4.5%	8<br>3.5%	3<br>1.6%	65<br>2.6%	0.113
INHR	13<br>5.2%	10<br>4.4%	5<br>1.8%	12<br>4.2%	16<br>5.9%	10<br>3.5%	8<br>3.1%	5<br>1.9%	4<br>1.8%	9<br>4.8%	92<br>3.6%	0.025
RIFR	0<br>0.0%	1<br>.4%	0<br>0.0%	1<br>.3%	1<br>.4%	2<br>.7%	3<br>1.1%	0<br>0.0%	0<br>0.0%	1<br>.5%	9<br>.4%	0.210
PZAR	2<br>.8%	1<br>.4%	8<br>2.9%	3<br>1.0%	6<br>2.2%	6<br>2.1%	6<br>2.3%	16<br>6.0%	11<br>4.8%	17<br>9.0%	76<br>3.0%	< 0.05
PolyR	6<br>2.4%	6<br>2.6%	2<br>.7%	4<br>1.4%	3<br>1.1%	6<br>2.1%	4<br>1.5%	7<br>2.6%	0<br>0.0%	3<br>1.6%	41<br>1.6%	0.158
MDR	8<br>3.2%	2<br>.9%	6<br>2.2%	8<br>2.8%	6<br>2.2%	10<br>3.5%	6<br>2.3%	7<br>2.6%	1<br>.4%	4<br>2.1%	58<br>2.3%	0.248
Total	248	228	276	288	273	284	261	266	227	188	2539	

*SMR (Streptomycin-resistant), INHR (Isoniazid-resistant), RIFR (Rifampicin-resistant), PZAR (Pyrazinamide-resistant), PolyR (Poly-resistant), MDR (Multi-drug-resistant), XDR (Extensively-drug-resistant)
