# Supplementary material for: Drug resistant Mycobacterium tuberculosis in Oman: resistance-conferring mutations and lineage diversity
Source: PeerJ. 2022 Jul 28;10:e13645. doi: 10.7717/peerj.13645 (PMC9339217; doi:10.7717/peerj.13645)
Supplement: Supplemental Information 3 [file peerj-10-13645-s003.pdf]

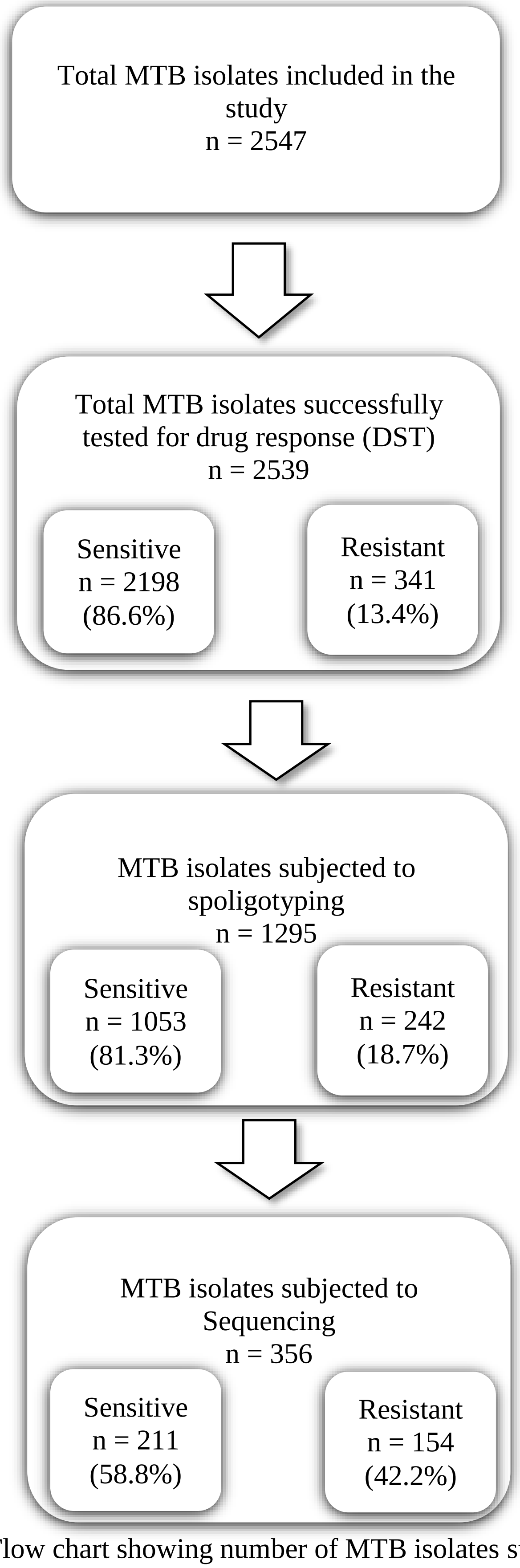

Supplementary Figure 1. Flow chart showing number of MTB isolates successfully analyzed at different stages of the study.
